# Human health risk assessment of heavy metals in drinking water sources in three senatorial districts of Anambra State, Nigeria

**DOI:** 10.1016/j.toxrep.2022.04.011

**Published:** 2022-04-15

**Authors:** Ugwu Chidiebere Emmanuel, Maduka Ignatius Chukwudi, Suru Stephen Monday, Anakwuo Ikechukwu Anthony

**Affiliations:** a Department of Human Biochemistry, Faculty of Basic Medical Sciences, Nnamdi Azikiwe University, Nnewi Campus, Nigeria; b Department of Medical Laboratory Sciences, Faculty of Health Sciences and Technology, Nnamdi Azikiwe University, Nnewi Campus, Nigeria

**Keywords:** Heavy metals, Water, Cancer, Hazard risk, Anambra State

## Abstract

Direct exposure to heavy metals (HMs) in drinking water beyond the allowable tolerable limit can adversely affect human health. The study evaluated the hazards (cancer and non-cancer) of HMs in drinking water for both children and adults based on hazard quotients (HQ) derived from the concentration of HMs in drinking water in Anambra State, Nigeria.

Eighty-one water samples were collected from 5 water sources (borehole, well, sachet water, harvested rain, and stream water) in 3 districts of Anambra State, and their concentrations of selected HMs [lead (Pb), cadmium (Cd), and mercury (Hg)] were analyzed by Atomic Absorption Spectrometry (AAS). The health risks were assessed based on the concentrations of HMs in the water samples ingested orally.

The concentrations of the HMs were higher than the permissible limits recommended by international agencies. The chronic daily intake (CDI) indices in the studied areas were highest for Cd. The CDI indices in the borehole, well, sachet water and, stream water samples were Cd>Hg>Pb for both populations. The CDI was higher in children compared to adults exposed to the same water sources. The hazard quotient (HQ) indices for HMs in the various water sources were in the order Cd>Pb>Hg for both populations. The hazard index (HI) of Pb was highest in rain water while that of Hg and Cd were highest in stream water for both adults and children. The incremental life cancer risk (ILCR) in the studied areas showed a higher risk for children than adults. Cadmium was a major risk factor and children at greater cancer risk than adults. Generally, the HQ and ILCR were greater than international standards with values for children higher than adults. The contribution of Cd towards HI and ILCR in all cases was significant.

This study showed the concentrations of the HMs in drinking water sources, and their attendant HQ and ILCR. These values were higher than the permissible limits set by international agencies. The results demonstrated enormously worrisome risks for children than adults.

## Highlights

• The concentrations of the heavy metals were above the permissible limits.
• Cadmium contributed highest to the chronic daily intake.
• The hazard index was greater than unity.
• Children were at greater cancer risk than the adults.

## Graphical Abstract

**Figure:**
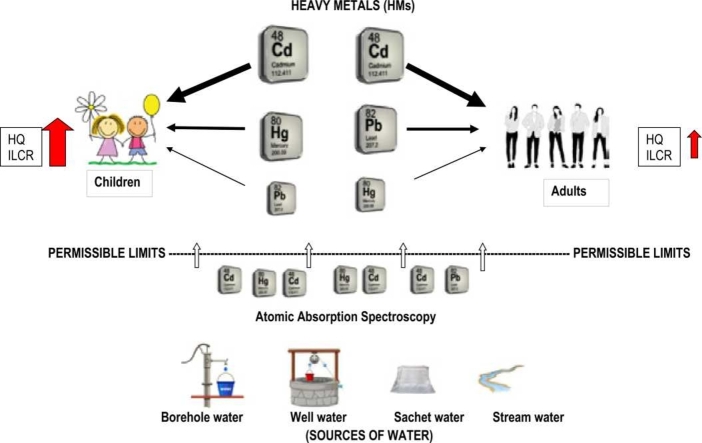

## 1 Introduction

Direct exposure to heavy metals (HMs) in drinking water beyond permissible limits has become a major public health concern, especially in the developing world. Anthropogenic activities causing the release of HMs from the naturally trapped sources into water sources have been identified [1]. The most common routes of human exposure to HMs in industrial and residential areas are dermal, inhalation and oral ingestions (food, water) [2]. These HMs can cause toxicity if their allowable levels are surpassed [3]. The HMs are non-biodegradable and may amass in the ecosystem reaching unsafe proportion for human health [4], [5]. Besides raw sources of water, water packaging materials have become major sources of contaminants in bottled and sachet water [3].

The impact of heavy metal contaminants in drinking water and the attendant health risks are important factors to be considered when evaluating drinking water quality [6], [7]. A proper risk assessment involves establishing the capacity of a risk source to introduce contaminants into the environment, determining the quality of risk agents that came in contact with the human, animal, and plant environment boundaries [8], and then quantifying the health implications of the contact or exposure [9]. Heavy metal entering the body through these routes could elicit carcinogenic and non-carcinogenic health risks [10]. Internal contact with some heavy metals is of concern from a health perspective [11]. The chemical form of a metal can influence its toxicity and buildup in human body [12]. For example, lead (Pb) in its inorganic and organic forms are absorbed to about 15% and 80% respectively when ingested [12]. While inorganic mercury (Hg) is toxic to the kidney, its organic form is a potent nervous toxicant [13] when ingested. Lead is an extremely toxic and may cause protracted health risks including headache, loss of appetite, birth deficiencies, mental retardation, hypertension, lung cancer, and renal damage [7]. Cadmium (Cd) is a known carcinogen and can elicit critical effects on the kidney and bone due to its preferential pattern of distribution in these organs [14], [15]. Mercury is lethal and might be connected with the development of joint ailments, a decline of mental status among others [16]. The potential health implications of several contaminants in an ecosystem can be estimated by assessing the potential hazard risk involved [17]. Many studies have used this strategy to assess the likely deleterious health risks of human exposure to contaminated water sources [18], [19], [20].

The health risk assessment is a good tool for assessing the link between the environment and human health that can be expressed quantitatively in terms of hazard degree [21]. Thus, this study evaluated the health risks associated with oral exposure to selected heavy metals in different water sources in Anambra State Nigeria.

## 2 Materials and methods

### 2.1 Study site

**Fig. 1:**
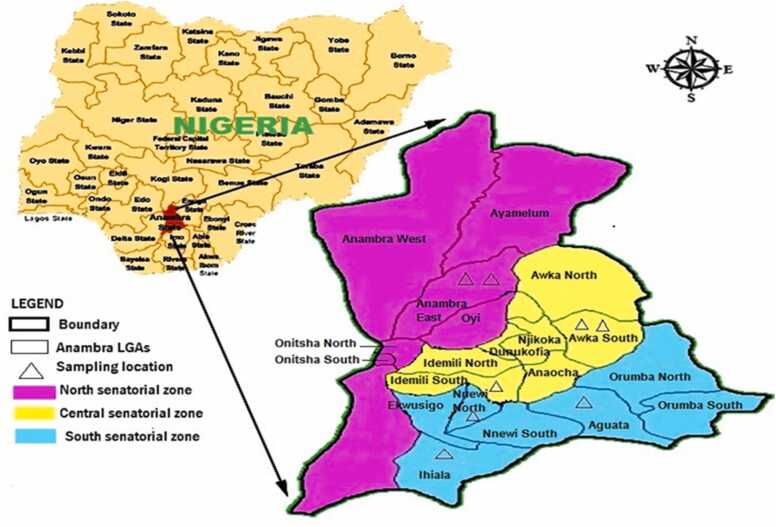
Sampling site in the three Senatorial districts.

This research was undertaken in the three Senatorial districts of Anambra State, Southeastern Nigeria. Anambra State lies geographically between Latitudes 6° 12 N, Longitude 6° 99 E, and 7° 00 W. The State consists of 21 Local Government Areas covering 3 Senatorial districts; Anambra Central, Anambra North, and Anambra South (Fig. 1).

(Source Google map).

### 2.2 Sampling technique

A stratified random sampling method was used in the selection of samples in the different Senatorial districts of the State. The sampling population was systematically arranged in a stratum when choosing the sampling points. In order to give equal representation, three sampling points were randomly selected in each Senatorial district.

### 2.3 Sample collection

Water samples were randomly collected from eighty one (81) sites which served as drinking water sources: 9 streams, 9 sachets, 18 boreholes, 18 wells, and 27 harvested rainwater. The sample points/sites of collection served as drinking water source. Harvested rainwater samples were collected from the run-off of galvanized roofing sheet from a plastic container on top of the roof in each of the three Senatorial districts of Anambra State Sampling was done from May – July 2015. Preliminary measures were taken following the standard guidelines [22] to avoid any possible contaminations. Harvested rainwater samples were collected in a plastic container placed on top of the roof of buildings. Water samples were collected by immersion of a sterile (5 mL) plastic universal container below the water surface for both streams and well samples. Borehole water samples were collected directly from the plastic tap supply outlet, after running the tap for about 2 min. The sachet water samples were collected from their respective production plants. The samples were taken to the laboratory immediately after collection and analyzed within seven days. Distilled water served as control.

### 2.4 Sample Analysis

The chemicals and reagents used in the study were of analytical grade (BDH Chemicals Ltd, UK). To ensure the removal of organic impurity from the samples and prevent interference in HM analysis, each water sample was acidified with concentrated nitric acid. One mL of nitric acid was added to 5mI of water sample and allowed to mix uniformly. Stock solutions (1000 mg/L) of Hg, Cd and Pb were prepared by dissolving in 1 L volumetric flask; 24.62 g, 1.63 g and 1.60 g of mercury sulphate decahydrate, cadmium chloride and lead nitrate respectively in 5% nitric acid for each metal. The mixture was shaken and the flask was made up to the 1 L mark with nitric acid for each metal. Calibrated solutions of the target metal ions were prepared from the standard stock by serial dilution. Calibration curve for each metal was prepared by plotting the absorbance of standards against their concentrations.

The acidified water samples were analyzed for the presence of mercury, cadmium, and lead respectively using atomic absorption spectrophotometer (Varian AA240) that uses air-acetylene flame [23]. The hollow cathode lamp of each corresponding element was the resonance line source. The acidified samples were analyzed in duplicates with the average concentration of the metal present extrapolated from the calibration curve. The wavelengths for determination of the elements were 309.30 mm, 228.80 mm, and 283.31 mm for mercury, cadmium, and lead respectively. The corresponding limit of detection (LOD) for Hg, Cd and Pb were 0.0010, 0.0020, and 0.0010 (ppm) respectively.

## 2 Human health risk assessment

### 2.1 Exposure assessment

To assess both non-cancer and cancer risks for children and adults, the chronic daily intake (CDI) of HMs, which represents the lifetime average daily dose (LADD) of exposure to a contaminant was used [1], [24]. The CDI of the HMs via oral ingestion was calculated using Eq. 1:

(1) CDI = (C x IR x EF x ED)/(BW x AT)

Where: CDI is the chronic daily intake (mg/kg/day); C is the concentration of the contaminant in water sample (mg/L); IR is the ingestion rate per unit time (1 L/day for a child and 2.2 L/day for an adult)[1]; ED is the exposure duration (6 years for a child and 30 years for an adult); EF is the exposure frequency (365 days/year); BW is body weight (15 kg for a child and 70 kg for an adult); AT is the average exposure time (for carcinogens, AT = 70 × 365 = 2550 days for both children and adults; for non-carcinogens, AT = ED x 365 = 2190 days and 10950 days for children and adults, respectively) [1]. The other variables for estimating human risk assessment through different pathways are listed in Table 1.

**Table 1:** Parameters used for estimating exposure assessment of heavy metals in drinking water.

Parameters	Unit	Value
Concentration of heavy metal	mg	–
Water ingestion rate (IR)	L/day	2.2
Exposure frequency (EF)	day/year	365
Average exposure time (adults) (AT)	days	10,950
Average exposure time(children) (AT)	days	2190
Exposure duration (adults) (ED)	years	30.0
Exposure duration (children) (ED)	years	6.0
Average body weight (adult) (Bw)	Kg	70.0
Average body weight (children) (Bw)	Kg	15
Oral reference dose (Cadmium)	mg/kg/day	0.0005
Oral reference dose (lead)	mg/kg/day	0.0003
Oral reference dose (mercury)	mg/kg/day	0.001
Cancer slop factor (lead)	mg/kg/day	0.0085
Cancer slop factor (cadmium)	mg/kg/day	6.3

Ref: [1], [10], [38], [42].

### 2.2 Non-Cancer risks

Non-cancer risks due to non-carcinogenic effects of HMs in drinking water were determined by the non-cancer hazard quotient using Eq. 2:

(2) HQ = CDI/RfD

Where: HQ is the non-cancer hazard quotient; CDI is the chronic daily intake (mg metal/kg/day); and RfD representing the chronic oral reference dose, that approximates the human population daily oral exposure level, plus delicate subpopulation which is probably to be without a significant risk of harmful effect through lifetime [25]. Potential risk to human health posed by exposure to multiple HMs was measured by the chronic hazard index (HI), which is the sum of all HQ calculated for each heavy metal [26]. A value of HQ or HI < 1 implies no significant non-cancer risks; a value ≥ 1 implies significant non-cancer risks, which increase with the increasing value of HQ or HI [27].

### 2.3 Cancer risk

Cancer risk is the hazard from a lifetime average dose exposure to 1 mg/kg body weight/day of a pollutant. Cancer risk was expressed in terms of incremental lifetime cancer risk (ILCR), which is the probability that one may develop cancer over a 70-year lifetime due to a 24-hour exposure to a potential carcinogen [28]. Cancer risk was calculated as the product of CDI (mg/kg/day) and cancer slope factor (CSF) measured in (mg/kg/day)^−1^ (see Eq. 3) [28]:

(3) ILCR = CDI x CSF

Where: ILCR = incremental life cancer risk; CDI = chronic intake (mg/kg/BW/day); CSF = cancer slope factor.

The total cancer risk as a result of exposure to multiple contaminants due to consumption of a particular type of water was assumed to be the sum of each metal incremental risk (∑ILCR). The United States Environmental Protection Agency (USEPA) considers the minimum or acceptable cancer risk for regulatory purposes within the range of 1 × 10^−6^ to 1 × 10^−4^[26].

### 2.4 Statistical analysis

The statistical analysis was done using SPSS 20 software (SPSS Inc., Chicago). Analysis of variance (ANOVA) was used to test whether or not significant differences existed between groups. Statistical significance was considered at p < 0.05.

### 2.5 Results and discussion

This study assessed the risk associated with insidious oral ingestion of three HMs; lead (Pb), mercury (Hg) and cadmium (Cd). The results summarized in Table 2 showed the mean concentrations of the HMs in the different drinking water sources from each Senatorial District. The comparative analysis of the mean HMs concentration in water sources showed significant differences among the different water sources in the 3 Senatorial districts.

**Table 2:** Concentrations (mg/l) of selected heavy metals in the various water samples from the Senatorial districts in Anambra State.

Anambra North					
Heavy Metals	BH	SW	HR	PS	StW
Cd	0.21 ± 0.03	0.20 ± 0.03	0.24 ± 0.03	0.23 ± 0.00	0.22 ± 0.03
Hg	0.07 ± 0.05	0.05 ± 0.04	0.16 ± 0.05	0.20 ± 0.18	0.35 ± 0.15
Pb	0.03 ± 0.02	0.05 ± 0.05	0.00 ± 0.00	0.00 ± 0.00	0.00 ± 0.00

Anambra Central					
Heavy Metals	BH	SW	HR	PS	St W
Cd	0.10 ± 0.05	0.20 ± 0.06	0.14 ± 0.04	0.15 ± 0.03	0.20 ± 0.02
Hg	0.08 ± 0.01	0.00 ± 0.00^a^	0.16 ± 0.01^c^	0.11 ± 0.11	0.14 ± 0.06
Pb	0.11 ± 0.12	0.12 ± 0.10	0.10 ± 0.10	0.00 ± 0.00	0.02 ± 0.01

Anambra South					
Heavy Metals	BH	SW	HR	PS	St W
Cd	0.28 ± 0.02^b^	0.32 ± 0.03	0.35 ± 0.04	0.34 ± 0.04^a^	0.28 ± 0.02^b^
Hg	0.38 ± 0.16^b^	0.20 ± 0.20	0.20 ± 0.21	0.20 ± 0.20^a^	0.38 ± 0.14^b^
Pb	0.00 ± 0.00^b^	0.11 ± 0.08^b^	0.34 ± 0.23	0.37 ± 0.08^a^	0.23 ± 0.06


Results are mean ± SD of triplicate readings. Values with different superscript a, b, c in a row are significant (P < 0.05). Cd = cadmium, Hg = mercury,Pb = lead, BH = borehole water, SW = shallow well water, HR = harvested rain, PS = processed sachet water and St W = stream water.

In the various water sources containing Pb, the levels of Pb ranged from 0.10 to 0.37 mg/L (Table 2), which were above the permissible limit of 0.01 mg/L [29]. Lead has the highest concentration in processed sachet water (0.37 ± 0.08) (P < 0.05) in Anambra South. The highest concentration of Pb from Anambra South may have resulted from high industrial activities of lead utilizing industries in the district. Lead can interfere with the normal enzymes function in the body. Lead toxicity can also damage the DNA and its consequent adverse neurological, haematological and nephrotoxic problems [30].

Water sources containing Hg recorded a concentration range of 0.05–0.38 mg/L (Table 2) which were far above the permissible contaminant level of 0.006 mg/L [29]. Stream water sources in the 3 Senatorial districts had the highest Hg contamination and Anambra South recorded the highest. The major source of Hg in the general population is through the consumption of fish and shellfish. Most of the Hg released from human activities is released into the air through fossil fuel combustion, mines, smelting, and solid waste combustion. Eventually, all Hg that is released in the environment will end up in soil or surface waters and may find their way to drinking water sources. By ingestion in drinking water, Hg exhibits its toxic effects by causing poor neurological development and immunodeficiency [30].

The results showed that the mean cadmium concentration range was 0.10–0.35 mg/L, which is significantly higher than the permissible limit of 0.003 mg/L [29]. Anambra South district recorded the highest Cd level and this was significantly higher in harvested rain and sachet water sources. The International Agency for Research on Cancer (IARC) classified Cd as carcinogenic among other health risk associated with its dietary intake that are related to damages to neurons, skeleton, and kidneys including cardiovascular disorders [30]. The higher values of these HMs above the recommended permissible limit showed the low-quality of water consumed by the residents and the associated health risk in the districts, especially in Anambra South.

The results for the chronic daily intake (CDI) (The average daily intake dose, ADI) for the ingestion pathway in the 3 Senatorial districts are shown in Table 3 for both adults and children. The results showed that the CDI values were slightly above the reference dose as recommended by USEPA or other international bodies. The ADD for Pb was highest in the water samples collected from Anambra South for both adults and children but least in Anambra North for both adults and children. Our results are in agreement with the report of Ayantobo et al.[31] and Ekere et al.[32]. The CDI values for Hg and Cd were equally highest from Anambra South District for both adults and children. CDI values were higher in children compared to adults exposed to the same drinking water sources in the three districts. The CDI indices for the HMs in the study areas were found to be in the order Cd > Pb > Hg (Pb=Hg) for adults and Cd > Hg > Pb for the children population.

**Table 3:** Chronic daily intake (CDI) or average daily intake (ADI) dose in different Senatorial district of Anambra State (mg/kg/day).

Senatorial district	Lead		Mercury		Cadmium	
	Adults	Children	Adults	Children	Adults	Children
Central	0.00314	0.01467	0.00157	0.00733	0.00440	0.02053
North	0.00063	0.00293	0.00409	0.01907	0.00723	0.03373
South	0.00691	0.03227	0.00691	0.03227	0.01037	0.04840

The CDI values in different drinking water sources are presented in Table 4. The CDI indices for the heavy metals in the borehole water, shallow well-water, processed sachet water, and steam water sources were found to be in the order Cd > Hg > Pb for both the adults and children populations. In harvested rain, the CDI value was found to be highest with cadmium followed by lead.

**Table 4:** Chronic daily intake (CDI) or average daily intake (ADI) in different drinking water sources (mg/kg/day).

Heavy Metals	Borehole water		Shallow well		Harvested rain water		Processed sachet water		Stream water	
	Adults	Children	Adults	Children	Adults	Children	Adults	Children	Adults	Children
Lead	0.00157	0.00733	0.00314	0.01467	0.00440	0.02053	0.00377	0.01760	0.00314	0.01760
Mercury	0.00220	0.01027	0.00566	0.02640	0.00377	0.01760	0.00471	0.02200	0.00629	0.02200
Cadmium	0.00629	0.02933	0.00723	0.03372	0.00754	0.03520	0.00754	0.03520	0.00849	0.03520

For Pb the CDI trend was in the order; harvested rain > processed sachet water > (shallow well = stream) > borehole water for adults and (stream = processed sachet water) > borehole > shallow well > harvested rain for children. The CDI values for Hg were highest in stream water (6.29 × 10^−3^) and least in borehole waters (2.20 × 10^−3^) for the adults while the trend reversed for the children. The results showed that the CDI values were high in children relative to adults and the highest CDI for Cd was from stream waters in the State. This may be due to the direct effects of effluents from the high industrial activities in the State. Our result on the CDI values of Cd is similar to the report of some previous studies [31], [32], [33]. Results from human and animal studies have shown that Cd may predispose humans to cancer [34], [35]. It is clear from the results that cadmium contributed most to the high CDI values observed in the study and could be a major health risk.

The hazard quotient (HQ) and hazard index (HI) for the HMs in the 3 Districts are presented in Table 5. The HQ values for the 3 HMs were higher in children compared to adults while the Anambra South District recorded the highest HQ for both children and adults. The values of HQ indices for the HMs in the Central and Southern districts for both adults and children were in the order Pb>Cd>Hg while the Northern district trend was Cd > Hg > Pb. The HQ values for the HMs were significantly greater than 1 and may indicate high carcinogenic/non-carcinogenic risk to residents of the State [36]. Likewise, the HI trend was South>Central>North for both populations (Table 5). The observed trend may not be unconnected with the high Pb and Cd producing waste industries in both the Southern and Central districts. The hazard quotients for the HMs which were greater than 1 signifies that the population would also experience non-cancer risks due to exposure to these HMs in drinking water.

**Table 5:** Hazard quotient and Hazard index in different Senatorial districts of Anambra State.

Senatorial district	Lead		Mercury		Cadmium		Hazard Index (HI)	
	Adults	Children	Adults	Children	Adults	Children	Adults	Children
Central	10.48	48.89	1.57	7.33	8.80	41.06	20.85	97.29
North	2.09	9.78	4.08	19.06	14.46	67.47	20.64	96.31
South	23.05	107.56	6.91	32.27	20.74	96.80	51.13	236.62
∑(HI)	92.62	430.22						

The HQ and HI for the heavy metals as calculated for the different water sources in this study are shown in Table 6. The HQ for the HMs was also higher in children than in adults for the various water sources. The values for the HQ indices for Pb were in the order; harvested rain>processed sachet water> (shallow well=stream)>borehole water for both adults and children while for Hg the HQ trend was stream>shallow well>processed sachet water>harvested rain. For Cd the HQ trend was stream>processed sachet water>shallow well>borehole. The HQ was > 1 in all the water sources used in the study. The HQ indices > 1 calculated for all the water samples presents an unacceptable risk for non-carcinogenic adverse effect especially as it concerns Cd. Cadmium contributed most towards the exposure to non-cancer risks in the exposed population followed by lead. Bamuwuwamye et al. [1] reported Pb to be a major contributor to non-cancer risks. A HQ value of 1 < HQ < 5 suggests a level of concern while a value of 10 < HQ < 100 demands further data collection [26]. The results from this report indicate that there was a need to further collect data for the HMs especially for Cd and Pb. It, therefore, means that health risk on long-term exposure is high and the non-cancer adverse effect is equally of concern and not be neglected. The hazard indices for children were higher compared to those of the adults implying that children could be more disposed to non-cancer risks than adults. Our results are in tandem with some previous observations [1], [26], [37].

**Table 6:** Hazard quotient and Hazard index in different water sources.

Heavy Metals	Borehole water		Shallow well		Harvested rain water		Processed sachet water		Stream water		Hazard Index (HI)	
	Adults	Children	Adults	Children	Adults	Children	Adults	Children	Adults	Children	Adults	Children
Lead (mg/l)	5.24	24.44	10.48	48.89	14.67	68.44	12.57	58.67	10.48	48.89	53.43	249.33
Mercury (mg/l)	2.20	10.27	5.66	26.40	3.77	17.60	4.71	22.00	6.29	29.33	22.63	105.60
Cadmium (mg/l)	12.57	58.67	14.46	67.47	15.09	70.40	15.09	70.40	16.97	79.20	74.17	346.13
∑(HI)											150.22	701.06

The results of the carcinogenic risk due to HMs exposure in drinking water sources are presented in Table 7, Table 8. The incremental life cancer risk (ILCR) via oral ingestion of Pb and Cd in the 3 districts showed a higher risk for children relative to adults (Table 7). The ILCR from Table 8 showed that Cd was a major contributor to cancer risk from the different sources of water supply and that children were at greater cancer risk than adults. The ILCR of Pb and Cd for both adults and children were 0.23 and 1.09 respectively for all the 5 different water sources. Apparently, Cd was a major cancer risk from the studied water sources. The United States Environmental Protection Agency (USEPA) proposed an acceptable ILCR range of 1.00 × 10^−6^ to 1.00 × 10^−4^ [38]. Based on recommendations of USEPA, the carcinogenic risk range for Pb is 1.3 × 10^−5^ - 3.74 × 10^−5^ and 6.23 × 10^−5^ - 1.75 × 10^−4^ for both adults and children respectively for all the different water sources. The carcinogenic risk range obtained for Cd was 5.35 × 10^−2^ - 3.96 × 10^−2^ and 1.85 × 10^−1^ - 2.49 × 10^−1^ for both adults and children (Table 8). There was no significant difference among the water sources in terms of cancer risks but there was a significant difference between Cd and Pb. Hence, the cancer risks emanating from Pb, Hg, and Cd in water sources from the 3 districts warrant urgent attention.

**Table 7:** Incremental Life Cancer Risk (ILCR) and Total Cancer Risk(TCR) in different Senatorial districts of Anambra State.

Senatorial district	Lead		Cadmium		Total cancer risk (TCR)	
	Adults	Children	Adults	Children	Adults	Children
Central	2.67 × 10^−5^	1.22 × 10^−4^	0.03	0.13	9.08 × 10^−5^	4.24 × 10^−4^
North	5.34 × 10^−6^	2.49 × 10^−5^	0.05	0.21	–	–
South	5.88 × 10^−5^	2.74 × 10^−4^	0.07	0.30	0.14	0.64
TCR = ∑(ILCR)					0.14	0.16

**Table 8:** Incremental Life Cancer Risk (ILCR) in different water sources in Anambra State.

Heavy Metals	Borehole water		Shallow well		Harvested rain water		Processed sachet water		Stream water		Total Cancer Risk (TCR)	
	Adults	Children	Adults	Children	Adults	Children	Adults	Children	Adults	Children	Adults	Children
Lead	1.34 × 10^−5^	6.23 × 10^−5^	2.67 × 10^−5^	1.25 × 10^−4^	3.74 ×^−5^	1.75 × 10^−4^	3.21 × 10^−5^	1.50 × 10^−4^	2.67 × 10^−5^	1.25 × 10^−4^	1.36 × 10^−5^	4.24 × 10^−4^
Cadmium	3.96 × 10^−2^	1.8 × 10^−1^	5 × 10^−2^	2.1 × 10^−1^	5 × 10^−2^	2.2 × 10^−1^	5 × 10^−2^	2.2 × 10^−1^	5 × 10^−2^	2.5 × 10^−1^	1.4 × 10^−1^	6.4 × 10^−1^
TCR = ∑(ILCR)	0.23	1.09										

A risk of 1.0 × 10^−3^ needs protective measures [39]. Related to this risk range, the results from this study demonstrated pronounced cancer risks for both adults and children from drinking water over a lifetime. Considering the carcinogenic risk on cumulative effects of the HMs studied, it was discovered that children are more at risk to carcinogenic risk as the ∑TICR was recorded to be above acceptable values (10^−6^ to 10^−4^) that regulatory authorities regard as unacceptable. The ILCR estimates the incremental increase in risk for the exposed populations over a lifetime but does not consider when cancer will occur [40]. Also, the constant variables used in calculating the risks imply that the carcinogenic and non-carcinogenic risks are directly proportional to the concentrations of HMs in the water samples. Apart from drinking water, efforts should be made to prevent early and insidious exposure to cancer-causing agents to prevent the occurrence of cancer in the future [41]. While there are regulations put in place to prevent an increase in the concentration of HMs in water, their implementations have not been vigorously pursued.

It is suggested that risk characterization be cumulative to take into account aggregate exposures to multiple compounds or mixtures causing similar toxicological effects [43]. This can be done by applying the Adversity Specific Hazard Index for Cummulative risk assessment [44] suitable for toxicants with multiple residues that can cause similar toxicological effects such as pesticides [2], [43] and polychlorinated biphenyls [30].

## 3 Conclusion

The study showed that the water sources in the Southern District had a higher concentration of these HMs that were higher than the permissible limits with children being at higher cancer risk than adults. In all cases, the contribution of cadmium towards HI and ILCR was significant.

## Authors statements

The revisions have been effected. We appreciate the comments of the Reviewers and hope to apply the new methods of risk assessment in our subsequent works.

## Declaration of Competing Interest

The authors declare that they have no known competing financial interest or personal relationships that could have appeared to influence the work reported in this paper.
